# Reflective and Reflexive Stress Responses of Older Adults to Three Gaming Experiences In Relation to Their Cognitive Abilities: Mixed Methods Crossover Study

**DOI:** 10.2196/12388

**Published:** 2020-03-26

**Authors:** Najmeh Khalili-Mahani, Atousa Assadi, Kate Li, Mahsa Mirgholami, Marie-Eve Rivard, Habib Benali, Kim Sawchuk, Bob De Schutter

**Affiliations:** 1 PERFORM Centre Concordia University Montreal, QC Canada; 2 McGill Centre for Integrative Neuroscience Montreal Neurological Institute McGill University Montreal, QC Canada; 3 Department of Communications Concordia University Montreal, QC Canada; 4 Armstrong Institute for Interactive Media Studies Miami University Oxford, OH United States

**Keywords:** silver gaming, serious games, stress, cognitive training, brain training games, exercise games, ICT

## Abstract

**Background:**

The gamification of digital health provisions for older adults (eg, for rehabilitation) is a growing trend; however, many older adults are not familiar with digital games. This lack of experience could cause stress and thus impede participants’ motivations to adopt these technologies.

**Objective:**

This crossover longitudinal multifactorial study aimed to examine the interactions between game difficulty, appraisal, cognitive ability, and physiological and cognitive responses that indicate game stress using the Affective Game Planning for Health Applications framework.

**Methods:**

A total of 18 volunteers (mean age 71 years, SD 4.5; 12 women) completed a three-session study to evaluate different genres of games in increasing order of difficulty (S_1_-BrainGame, S_2_-CarRace, and S_3_-Exergame). Each session included an identical sequence of activities (t_1_-Baseline, t_2_-Picture encode, t_3_-Play, t_4_-Stroop test, t_5_-Play, and t_6_-Picture recall), a repeated sampling of salivary cortisol, and time-tagged ambulatory data from a wrist-worn device. Generalized estimating equations were used to investigate the effect of session×activity or session×activity×cognitive ability on physiology and cognitive performance. Scores derived from the Montreal Cognitive Assessment (MoCA) test were used to define cognitive ability (MoCA-high: MoCA>27, n=11/18). Kruskal-Wallis tests were used to test session or session×group effects on the scores of the postgame appraisal questionnaire.

**Results:**

Session×activity effects were significant on all ambulatory measures (χ^2^_10_>20; *P*<.001) other than cortisol (*P*=.37). Compared with S_1_ and S_2_, S_3_ was associated with approximately 10 bpm higher heart rate (*P*<.001) and approximately 5 muS higher electrodermal activity (*P*<.001), which were both independent of the movement caused by the exergame. Compared with S_1_, we measured a moderate but statistically significant drop in the rate of hits in immediate recall and rate of delayed recall in S_3_. The low-MoCA group did not differ from the high-MoCA group in general characteristics (age, general self-efficacy, and perceived stress) but was more likely to agree with statements such as *digital games are too hard to learn*. In addition, the low-MoCA group was more likely to dislike the gaming experience and find it useless, uninteresting, and visually more intense (χ^2^_1_>4; *P*<.04). Group differences in ambulatory signals did not reach statistical significance; however, the rate of cortisol decline with respect to the baseline was significantly larger in the low-MoCA group.

**Conclusions:**

Our results show that the experience of playing digital games was not stressful for our participants. Comparatively, the neurophysiological effects of exergame were more pronounced in the low-MoCA group, suggesting greater potential of this genre of games for cognitive and physical stimulation by gamified interventions; however, the need for enjoyment of this type of challenging game must be addressed.

## Introduction

### Background

Playing digital games is quickly becoming a common pastime for many older adults [1,2]. Games are pleasurable activities when they offer a balanced mixture of challenge, reward, and competition. Playing games together is a social activity that may mitigate feelings of isolation. Playing games alone offers relaxation and distraction. Digital games have several potential advantages: they may offer higher sensory stimulation by their visual and sound effects; they may challenge executive and motor skills, visual attention, and speed of reaction in decision making; and they can also be customized in interface or level of difficulty. Research indicates that many older adults are onboard with *serious* digital play [3-7]. To develop serious games (ie, games in which the intention is to benefit tangibly from game play and attempt to deliver cognitive, emotional, or rehabilitation training to older adults) is a growing trend [8-15].

Although emerging data suggest that digital playing can improve cognitive, physical, and emotional health in older adults [16-23], the results are not conclusive. Currently, there are two dominant research streams addressing the complexity of developing serious games for seniors: (1) game designers evaluate the effectiveness of a game in terms of accessibility and meaningful and enjoyable play [6,24-32], and (2) health researchers focus mainly on their cognitive [33-36] or specific motor-related effects [19,25,37-49] in controlled trials. An empirical framework to evaluate the efficacy of different game-based health interventions that bridges these two fields is much needed [50,51].

At present, the inadequate design of computer games and low accessibility of game technologies are the major impediments to their adoption by seniors [42,52,53]. Successful games foster time-on-task and promote more effective learning because they raise motivation and arouse players’ interest, for instance, by providing instantaneous and informative feedback, intrinsic rewards, and features that allow players to self-assess and adjust the levels of game difficulty to their skills [54]. These neurological signals may transfer to positive psychological effects such as enjoyment and challenge resulting from gameplaying [55]. Several studies have shown that in general, more seniors prefer the ease and pleasure of casual games (preferably with an intellectual component) over more cognitively demanding action games [3,56,57]. However, interindividual variations in game perception and motivation are important factors that engender the game-playing experience [58,59]. Thus, while digital games are increasingly prevalent among the seniors who are offered access to digital devices, many seniors are not familiar with or accustomed to digital game playing. As such, the mere promise of functional enhancement through such activities may not suffice to motivate them to overcome the barriers of unfamiliarity with the technology, if they have access to that technology. Inserting a *for-health* computerized intervention, even if ludic, may still introduce stress either because it seems too difficult to master and thus underlines their disabilities, or because it is an unfamiliar and burdensome imposition on their lifestyle. Therefore, it is important to ask whether the adoption of proscribed gamified interventions for older adults may be too stressful.

### Objectives

**Figure 1:**
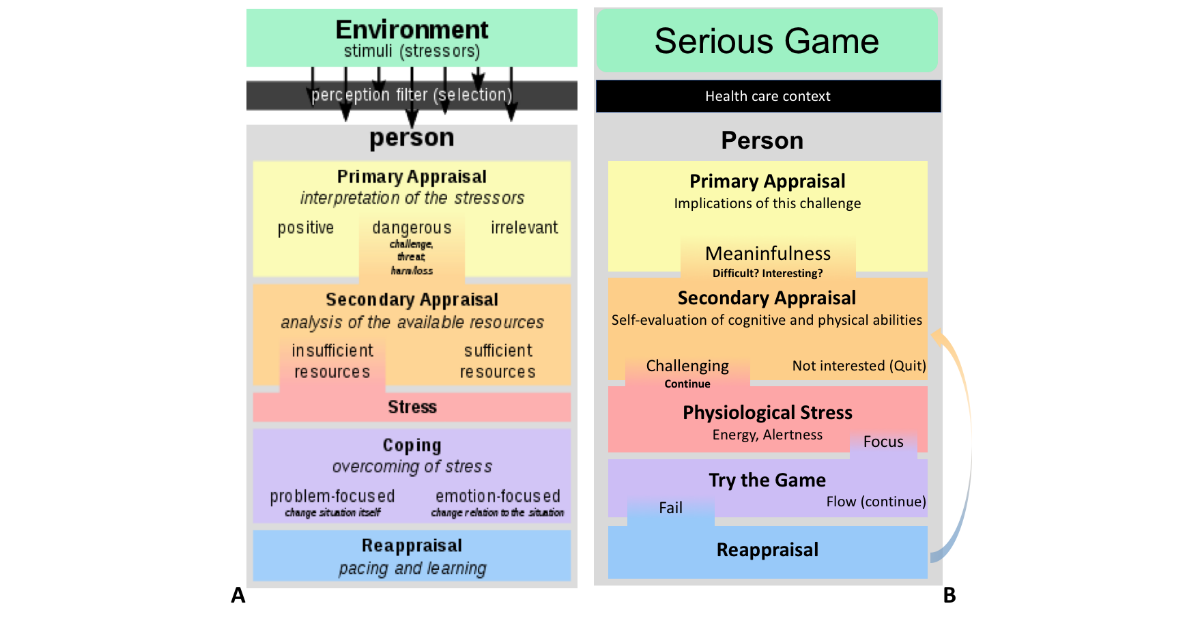
(A) Diagram of Lazarus and Folkman’s Transactional Theory of Stress (Source: Wikipedia, Philipp Guttmann), (B) our adaptation of Lazarus’s model to be tested in the Affective Game Planning for Health Applications framework.

This study aimed to address the question on whether the adoption of proscribed gamified interventions for older adults may be too stressful. To that end, a hybrid data/model-driven mixed methods framework for Affective Game Planning for Health Applications (AGPHA) was proposed [60]. AGPHA builds on the theory of appraisal and coping by Lazarus (summarized in Figure 1) and emphasizes that motivational, relational, and cognitive factors determine individual differences in perception of, and ability to cope with, new challenges (for an ontology by Lazarus, see [61]). These differences may manifest as quantifiable variations in the stress system of the body, including marked changes in cortisol response and autonomic signals such as heart rate (HR), skin conductance, and gut reflexes. These physiological reactions are the body’s nonspecific response to the perception of stress [62], and usually the intensity of these responses depends on an individual’s biological and psychological coping resources [63].

Figure 1 shows our adaptation of the theory by Lazarus [61]. When presented with a challenge, an individual’s primary appraisal determines its relevance, threat, and benefits. If it is irrelevant, the individual can ignore it. If it is threatening, they have to respond to it. If the challenge is beneficial, they may choose to respond to it. In case of games for health, the challenge is not threatening to one’s health, but if the games are introduced in the context of health care or cognitive fitness, they can be challenging to an individual’s self-esteem because the player may feel pressured to learn the game and perform well. How an individual responds to the game challenge will depend on the individual’s coping style and their cognitive, emotional, or physiological reserves. If they have a negative attitude toward the game, they might avoid it, or feel stressed by trying it. However, if they have a positive attitude, or an investment (such as expectation that it will improve cognitive function), then they will try to learn and overcome the challenges of the game. This is when the player enters the secondary stage of appraisal. In the secondary stage of appraisal, if the challenge is feasible, then the player may experience what is termed as *flow*; otherwise, it becomes *stressful*. Again, at the secondary appraisal, the personality and cognitive and physical reserves of the individual play a role in moderating the magnitude of the stress response and the behavioral outcome (in this case, playing further or giving up).

In the context of gamified strategies for cognitive training or testing, the complex relationship between stress and cognition deserves attention. It is well established that physiological stress responses modulate the function of central cognitive structures such as the hippocampus and the prefrontal cortex [64,65]. Stress interferes with learning through myriad cognitive processes, especially attention, reward processing, and decision making [66,67], which are important for habitual and complex activities that define an individual’s adaptation strategies in the course of life. Actual or perceived decline in cognitive abilities can influence a senior’s primary and secondary appraisal of the game and discourage them to try. Diminished cognitive abilities can make the game more challenging and thus lead to more pronounced physiological stress responses, which might cause health-related side effects that need to be monitored or avoided. It is also possible that excessive game stress would lead to distraction or memory failure, thereby impeding learning and skill development, which are important for motivation to replay.

Therefore, in evaluating whether games are stressful or not, AGPHA asks three central questions:

1. What are the characteristics of individuals who choose to play?
2. Is the playing experience quantifiably stressful?
3. Which intra- or intersubject factors (eg, appraisal, cognitive abilities, health status) predict variations in game-related stress, game-learning, and retention?

### Study Aims and Hypotheses

In this study, we provide an example of deploying the AGPHA framework to investigate the relation between game stress and cognitive abilities of the senior game players, introduced to three unfamiliar gaming experiences. In this study, we have tested the following hypotheses:

1. Playing unknown digital games will be stressful to older adults.
2. Game difficulty and general cognitive abilities predict a quantifiable increase in reflexive (ie, variations caused in physiological or neuropsychological function) and reflective (ie, subjective evaluation of the postgame experience) stress responses.

This study includes the following elements: (1) a survey element to allow us to understand the biases related to individual characteristics that attract older adults to the topic of gamified cognitive enhancement, (2) a within-subject factor (three genres of presumably cognitive enhancing games: brain training, car racing, and exercise gaming) that allows us to introduce variations in the phenomenology of gaming experience, (3) a between-subject factor (cognitive ability determined by the Montreal Cognitive Assessment [MoCA]) that allows us to investigate intersubject variations in game experience, and (4) two categories of reflexive (cortisol, ambulatory signals, and neuropsychological tests) and reflective (postgame evaluation of the game-play experience based on intrinsic motivation) outcome measures that would allow us to compare the sensitivity of objective biomarkers against subjective ratings.

## Methods

### Participant Recruitment

We adopted a snowball survey method to recruit potential participants and assess the characteristics of seniors who showed interest in the topic of gamified approaches to cognitive enhancement. With approval from the institutional review board, we targeted a general mailing list of the PERFORM Centre; this list consisted of individuals interested in volunteering for preventative health studies. We invited individuals older than 65 years to participate in *Finding Better Games for Older Adults: An Objective Assessment of Interactions Between Appraisal, Arousal and Cognitive Benefits of Electronic Playing.* The recruitment period lasted approximately 2 months. The survey asked questions about how they evaluated game-related activities in relation to a range of other activities (eg, gaming vs exercising, or talking with people rather than playing). The survey also included the General Self-Efficacy Scale (a 10-item scale) [68]; University of California, Los Angeles (UCLA) Loneliness Scale [69]; and Perceived Stress Scale (9-item scale [70]) to control for confounding chronic stress or tendency to become more easily stressed. We also asked participants to self-evaluate their state of mental and physical wellness as *good*, *bad*, or *could be better*.

### Study Sample

Those who completed the survey and could commit to three 120-min tests in our research facility were invited to participate in the follow-up experiments. On the basis of previous work [71], we estimated a sample of 20 participants sufficient to provide pilot data with 80% power to detect a difference of 0.17 (log [Cortisol nmol/mL]) in response to a psychological stress challenge between the study arms with an SD of 0.26 and one-tailed paired *t* test (*P*=.05, where more difficult games were expected to cause higher stress). We performed medical screening using the Physical Activity Readiness Questionnaire (PAR-Q+) to rule out hazards such as movement disorders that would increase the risk of falling, epileptic seizures, and visual impairments that would prohibit participants from playing digital games safely. Participants were required to be capable of walking continuously for 20 min; literate; and able to watch a television, a computer, and an iPad screen. Before participation, they signed an informed consent approved by the institutional ethics review board. They were free to discontinue the study at any moment. No financial compensation was offered.

### Experimental Design

Aim 1 was to determine whether novel gaming experiences induce a psychophysiological stress response, and whether there is a relation between appraisal of the game experience and psychophysiological measures. Aim 2 was to determine whether differences in cognitive abilities predict variations in game-related stress. Cognitive abilities were assessed by MoCA Original 7.0, a 30-point standardized outcome measure that is used clinically to screen for milder forms of cognitive impairment [72].

Following the AGPHA framework, we collected multifactorial data to create a comprehensive profile of the emotional, cognitive, and biological characteristics of the participants. Table 1 summarizes data that we collected in the study. A semistructured interview—to assess the challenge, excitement, enjoyment, goals, and likelihood to play and replay a game—was also performed, but those results are beyond the scope of this paper and will be presented separately.

**Figure 2:**
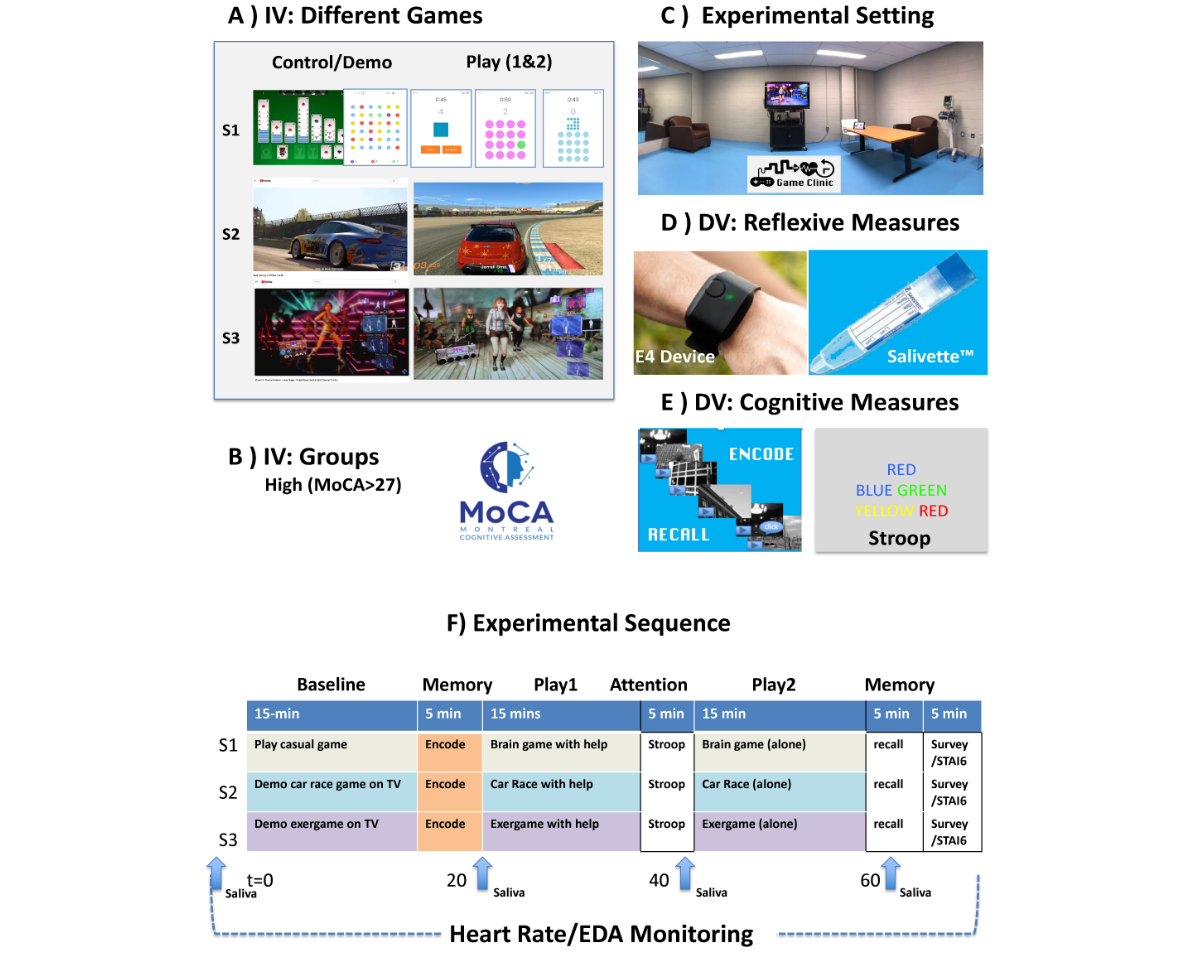
Experimental methods and procedure. (A) Different games that were used; (B) Montreal Cognitive Assessment tool was used as a predicting factor; (C) the experiment room, a small studio located at PERFORM Centre’s Gym; (D) devices used for measurement. DV: dependent variable; EDA: electrodermal activity; IV: independent variable; MoCA: Montreal Cognitive Assessment.

The experimental design was a repeated measures crossover study (Figure 2). Participants were fully informed about the intention of the study, and no deception was used:

We would like to hear how you evaluate different games which are suggested to enhance cognitive function. We want to know how you appraise them in terms of fun, difficulty, or benefits. We will also collect physiological data using an arousal-sensing device that you will be wearing on your wrist and from saliva samples that let us know if the game stressed your body.

Because this was a repeated measures study, we expected that increased familiarity with our experimental setting would reduce their overall stress. For this reason, we presented the games in increasing degrees of complexity and difficulty. Identical functional assessments (cognitive tests) were incorporated to control for learning effects. This design, accounting for habituation, allowed us to use the first (and presumably the simplest game) as a reference against which to compare gradual changes in game appraisal and familiarity with the game.

**Table 1:** Study variables.

Variable category and variable type		Screening	Baseline	Brain training	Car race	Dance
**Characteristics**						
	Demographics	✓	N/A^a^	N/A	N/A	N/A
	UCLA^b^ Loneliness Index	✓	N/A	N/A	N/A	N/A
	Perceived Stress Scale	✓	N/A	N/A	N/A	N/A
	General Self-Efficacy	✓	N/A	N/A	N/A	N/A
	Self-assessment of mental/physical health	✓	N/A	N/A	N/A	N/A
	Prestudy appraisal of game (IF^c^)	✓	N/A	N/A	N/A	N/A
**Cognitive**						
	Montreal Cognitive Assessment (IF)	N/A	✓	N/A	N/A	N/A
	Picture Encode/recall (DV^d^)	N/A	N/A	✓	✓	✓
	Stroop (DV)	N/A	N/A	✓	✓	✓
**Biometrics**						
	Heart rate and heart rate variability (DV)	N/A	✓	✓	✓	✓
	Electrodermal activity (DV)	N/A	✓	✓	✓	✓
	Accelerometer (CV^e^)	N/A	✓	✓	✓	✓
	Saliva cortisol (DV)	N/A	✓	✓	✓	✓
**Subjective**						
	Postgame appraisal survey (DV/IV^f^)	N/A	N/A	✓	✓	✓
	STAI-6^g^ (DV)	N/A	N/A	✓	✓	✓

^a^N/A: not applicable.

^b^UCLA: University of California, Los Angeles.

^c^IF: independent factor.

^d^DV: dependent variable.

^e^CV: control variable.

^f^IV: independent variable.

^g^STAI: Spielberger State-Trait Anxiety Inventory.

### Procedures

The study took place between 10 AM and 4 PM. Participants were asked to refrain from eating and drinking sweetened or caffeinated beverages or engaging in strenuous physical activity for at least 2 hours before their appointment at PERFORM. Each session was completed on separate days, with 3 to 7 days between visits. Each visit lasted about 2 hours. The details of the experimental procedure are presented in Multimedia Appendix 1. Briefly, *session 1* served as the baseline as we introduced players to cognitive training games played on iPAD (MindGame, by Tom Lake). In *session 2*, we introduced a visually and cognitively more complex game, using an iPad (Real Racing 3, v 5.4.0, Electronic Arts Inc), which allowed players to steer the car using the gyroscopic features of the iPad and thus required minimal efforts in learning the control buttons. In *session 3*, we introduced an exergame (Dance Central by Harmonix, MS Studios). In this game, players copy the moves of a virtual choreographer while the motion tracking Kinect evaluates them against the queued movement.

### Outcome Measures

#### Postgame Appraisal

To study the relation between the participant’s subjective appraisal of the gaming experience, we administered an exit survey at the end of each session, which was loosely based on the intrinsic motivation inventory and tapped into the question of enjoyment (*The experience was enjoyable, The experience was interesting*), competence (*The game was difficult*), tension (*The experience was stressful; I found this to be a frustrating experience. These games are visually intense*), choice (*I liked to play this game again; I will play this game again. I did not like this experiment*), and value (*This game help improve my mental wellness, I think this game is useless; These games are cognitively stimulating*). Instead of a 7-choice scale to measure degrees of satisfaction, we decided to implement a 4-choice response (definitely disagree, somewhat disagree, somewhat agree, and definitely agree), ranging from –2 to 2, but we allowed participants to add comments if they did not know the answer. We coded these responses to 0 or *I don't know*.

#### Saliva Cortisol

Saliva cortisol is often measured as a biomarker of a latent psychological modulation to the stress system [73]. Cortisol production displays a typical diurnal rhythm with a peak in the morning hours (8-10 AM), followed by a periodic pattern consisting of ultradian oscillations (20-120 min periods) [74,75]. We therefore ensured to schedule the visits at the same time for each individual, and we timed the experiment to avoid measuring the sharp rise in the awakening cortisol. Free circulating cortisol was assessed from saliva, collected using the Salivette device (Figure 2). The samples were frozen at −80°C, centrifuged at 4°C for 10 min, and analyzed using luminescence-immunoassay (IBL International) using enzyme-linked immunosorbent assay (at PERFORM Centre). The intra- and interassay coefficient variability was less than 12% and 5%, respectively.

#### Electrodermal Activity and Heart Rate Variability

Since 1970s, the electrodermal activity (EDA) and the heart rate variability (HRV) have been used as proxy markers of sympathetic autonomic response to arousing or stressful stimuli, finding successful application in laboratory experiments involving human-computer interface and games [76-82]. We used the E4 device (Empatica, Inc), a light wrist-wearable device for continuous monitoring of the physiological signals. The E4 band (Figure 2) was properly fit on the wrist of the nondominant arm. This device is equipped with 4 sensors: (1) photoplethysmography sensor to measure cardiac activity, (2) EDA sensor to measure skin conductance as a marker of *arousal* and *stress*, (3) 3-axis accelerometer (ACC) to measure the amount of movement along the x-, y-, and z-axes, and (4) optical thermometer to measure skin temperature.

To facilitate the replicability of our study, we downloaded preprocessed data from the Empatica data portal. We used our in-house MATLAB software on these data to extract data for each activity (by averaging signals’ amplitudes normalized to the duration of different activities). An *activity* denotes a specific interval measured from the onset of each experimental step. Activities were delimited using a *tag* button on the E4, which was pressed every time we administered a new task. A research assistant also kept track of detailed timing of the experiment. Because EDA is sensitive to the activity of sweat glands, the room temperature was maintained at 23°C. However, we also collected data from the temperature sensor and ACC on the E4 device to rule out confounding effects related to thermal or movement noise.

#### Stress-Sensitive Cognitive Tests

The triggering of physiological signals to stress serves the metabolic modulation of reward processing [83] and attention [84] systems that help individuals initiate an appropriate adaptive response to stress; thus, it can interfere with cognitive functions performed during or immediately after stress. We included a set of simple cognitive tasks to assess whether, despite the potential learning effect due to repeated nature of the design, increasing game difficulty would affect performance in cognitive tasks. The first set of cognitive tests used a picture encode/delayed recall memory test, which we have previously shown to be sensitive to stress modulations of the hippocampal function both under tasks [71] and under stress-inducing drug conditions [85]. In addition, we administered a simple Stroop word color test to assess an individual’s cognitive processing speed and their selective attention accuracy. The Stroop test has been shown to be sensitive to physical exertion and cognitive challenge [86,87]. All cognitive tests were presented at a minimal level of difficulty (with 3-second delay between stimuli) to ensure these tasks did not introduce added stress.

The encode/recall test consisted of two parts. In the first part (the encode condition), the participant was presented with 50 black and white images of random scenes, places, people, and objects. Each image stayed on screen for 3 seconds; thus, the participant had time to commit it to memory. This set of 50 pictures included 14 unique pictures and 36 nonunique pictures that were shown more than once. The participant was asked to press *m* as soon as they saw a familiar picture. They were told to remember these pictures for a recall test at the end of the session. In the second part (delayed recall), 45 black and white images were shown. This set included 15 pictures from the encode session and 30 new pictures. Participants were asked to press *m* as soon as they detected a previously seen picture. In both sessions, each picture remained on the screen for 3 seconds. If no answer was provided, the screen would proceed to the next picture. This test was implemented in E-Prime, and we recorded the number of hits, misses, and false alarms, both for the encode and the recall conditions. We had designed 3 sets of unique encode/recall pairs. In other words, none of the pictures seen in session 1 were repeated in the pictures presented in sessions 2 or 3.

We also wanted to measure the reaction time (RT) to the Stroop effect, that is the time it takes a player to process the incongruency between the color of a word read, and the color represented by the word. At each trial of this test, a brief training session was held to ensure the players understood the test objectives. Following the training session (1 min), a randomly selected color word (red, yellow, blue, or green) was presented to the participants. The color of the word on the screen may or may not have been congruent with the word itself. The stimuli (50) were randomly selected from a stack of 320 cards (80 of which were incongruent). The participants were asked to press a colored key on the keyboard, which was associated with the color of letters of the word, as fast as they could. In our implementation, we provided immediate feedback on whether the participants provided the correct answer or not. The participants were told that their scores would depend on how fast they responded to each card, but the time interval between cards was set to 5 seconds. The test was implemented in E-Prime, and we recorded the number of correct responses and the RT to correct response.

### Statistical Analysis

Our research questions were as follows:

- Can we detect within-subject game-related (ie, Session) differences in appraisal, physiological patterns over time (ie, ti: Activityi), and cognitive measures (immediate and delayed recall, and Stroop RT to correct)?
- Do differences related to cognitive abilities (measured by MoCA) predict differences in outcome measures for different sessions (S1: Brain training; S2: CarRace; and S3: Exergame) and activities (t1: Baseline; t2: Encode; t3: Play1; t4: Stroop; t5: Play2; t6: Recall)?

To examine the effect of *session*×*activity* in each outcome measure, we used generalized estimating equations (GEEs) with a robust estimator and an autoregression working correlations matrix. GEE is a special form of the general linear model that is recommended for longitudinal repeated measurements of within-subject data over time [88]. In all GEE models, *S_1_* and *t_1_* were used as within-subject references. For the group comparisons, all physiological factors were normalized to the within-subject, within-session baseline measures. For tests that included ambulatory measures as a dependent variable (EDA, HR, and HRV), we controlled for movement (measured from ACC) and duration of each activity. All results were plotted (mean and SEM), and post hoc comparisons were reported with least significant difference correction.

To explore differences in game appraisal between sessions, or between groups, we performed nonparametric tests (Spearman correlation and Kruskal-Wallis) on Likert scales. Results were plotted in terms of response frequency to each appraisal question to visually illustrate game- or group-related differences in the patterns of attitude toward games.

All statistical analyses were performed with SPSS (version 24 for Mac; IBM Corp). *MoCAGroup*×*Session*×*Activity* plots (mean, SEM) were generated with Prism (version 8 for Mac; GraphPad Inc).

## Results

### Who Wants to Play?

In total, 42 adults meeting the age criteria (>65 years) completed the survey, 19 of whom volunteered to enroll in the experimental study. Only 1 participant dropped out of the experimental phase after the first visit because of extreme physical discomfort that forced her to eat and drink (thus invalidating the cortisol sampling). Differences in participant characteristics are presented in Table 2. No significant group differences were observed (all *P*>.5).

**Figure 3:**
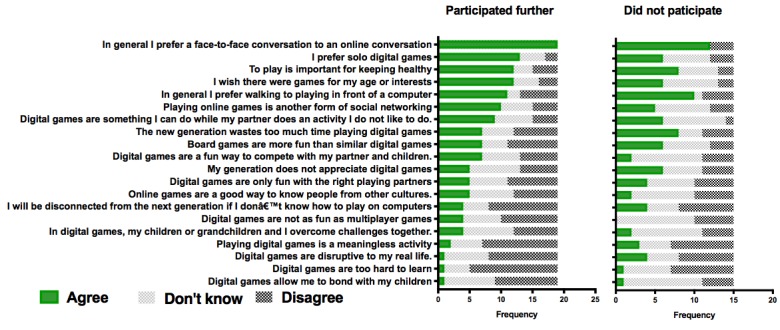
Participants’ appraisal of gaming before enrollment in the study.

Figure 3 summarizes the game attitude distribution in our sample. The response frequencies are ranked based on the largest number of favorable (*I agree*) responses to a given question in the sample who completed the study. Overall, those who completed the study and those who did not join the experiment after the screening had a positive attitude toward games; however, those who did not participate were not in disagreement but more frequently gave *I don’t know* responses to various game-related questions. Kruskal-Wallis tests showed that those who participated in the experimental phase gave more positive responses to the following items: *In general, I prefer face-to-face conversations to an online conversation* (χ^2^_1_=6.7; *P*=.01); *I prefer solo digital games* (χ^2^_1_=4.1; *P*=.04); *I wish there were games for my age or interest* (χ^2^_1_=4.2; *P*=.04). The participants more frequently disagreed with the following items: *Digital Games are too hard to learn* (χ^2^_1_=5.3; *P*=.02) and *Digital games are disruptive to my real life* (χ^2^_1_=2.8; *P*=.09).

**Table 2:** Participant characteristics.

Characteristics		Participated in experiment	Did not participate further
**Gender, n**			
	Male	6	10
	Female	13	13
	Other	0	0
**Mental health, n**			
	Good	17	13
	Could be better	2	2
**Physical health, n**			
	Good	14	10
	Could be better	5	5
Age (years), mean (SD)		70.47 (4.49)	69.70 (4.20)
Education (years), mean (SD)		16.26 (3.68)	16.22 (2.58)
Generalized Self-Efficacy, mean (SD), maximum n=40		33.79 (2.80)	32.42 (4.77)
Perceived Stress Scale, mean (SD), maximum n=40		17.78 (3.83)	18.46 (3.83)
Loneliness Index, mean (SD), maximum n=80		11.21 (11.36)	18.8 (15.54)

### Effects of Game Type on Reflexive Outcomes

#### Physiological Response

**Figure 4:**
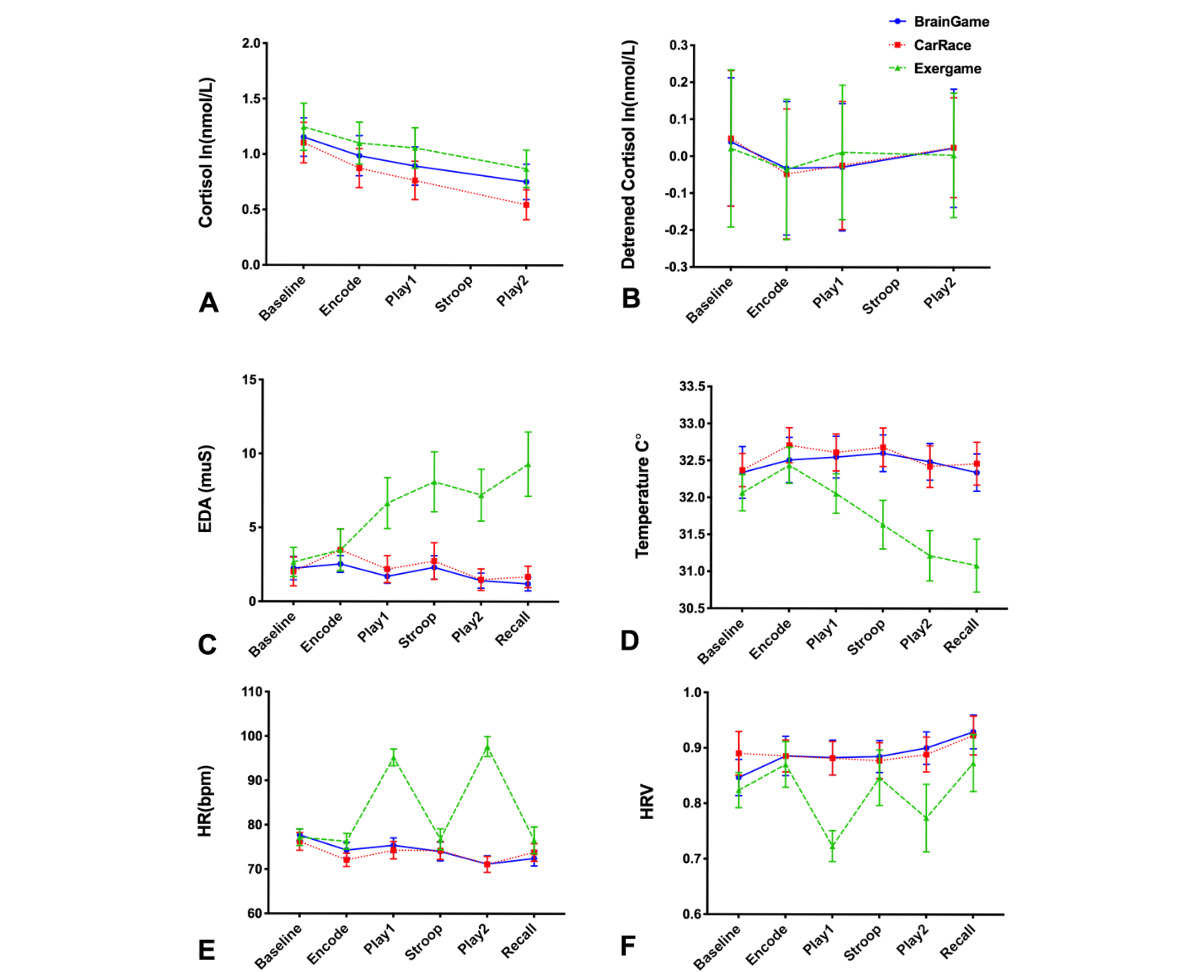
The profile of physiological signals changes over time. The only immediate significant game-related change was observed in heart rate and heart rate variability variables. HR: heart rate; HRV: heart rate variability.

Figure 4 illustrates the effects of games and activities on different signals measured during the experiment (mean, SEM). Session by activity interactions had a significant effect on EDA (χ^2^_10_=26; *P*=.004), HR (χ^2^_10_=24.5; *P*=.006), HRV (χ^2^_10_=96; *P*<.001) but not on Cortisol (χ^2^_6_=6.5; *P*=.37). In all sessions, we observed the highest levels of cortisol at the baseline, followed by an exponential decline in cortisol levels. Detrending the cortisol levels to remove this decline did not change the outcome of the statistical inference.

The main effect of *Session* was significant for EDA (χ^2^_2_=20.6; *P*<.001)*,* HR (χ^2^_2_=23.4; *P*<.001), and HRV (χ^2^_2_=33.3; *P*<.001). The effects of control variables (acceleration and duration) were not significant on any of the outcome measures (albeit a trend was observed for a positive association between acceleration and HR (*P*=.08). There were no significant differences between S_1_ and S_2_, but EDA in S_3_ was significantly higher than that in S_1_ (mean difference 4.3 muS; *P*<.001; 95% CI 2.77 to 5.9) and S_2_ (mean difference 3.97 muS; *P*<.001; 95% CI 2.4 to 5.5), expectedly as a result of physical activity. The HR in S_3_ was higher than that in S_1_ (mean difference 10.02 bpm; *P*<.001; 95% CI 7.5 to 12.2) and S_2_ (mean difference 9.6 bpm; *P*<.001; 95% CI 7.2 to 12 bpm) and the previous two sessions, but the difference between S_1_ and S_2_ was not significant. The main effect of *Activity* was significant only for HR (χ^2^_5_=28.8; *P*<.001) and HRV (χ^2^_5_=79.3; *P*<.001)*.*

#### Cognitive Performance

**Figure 5:**
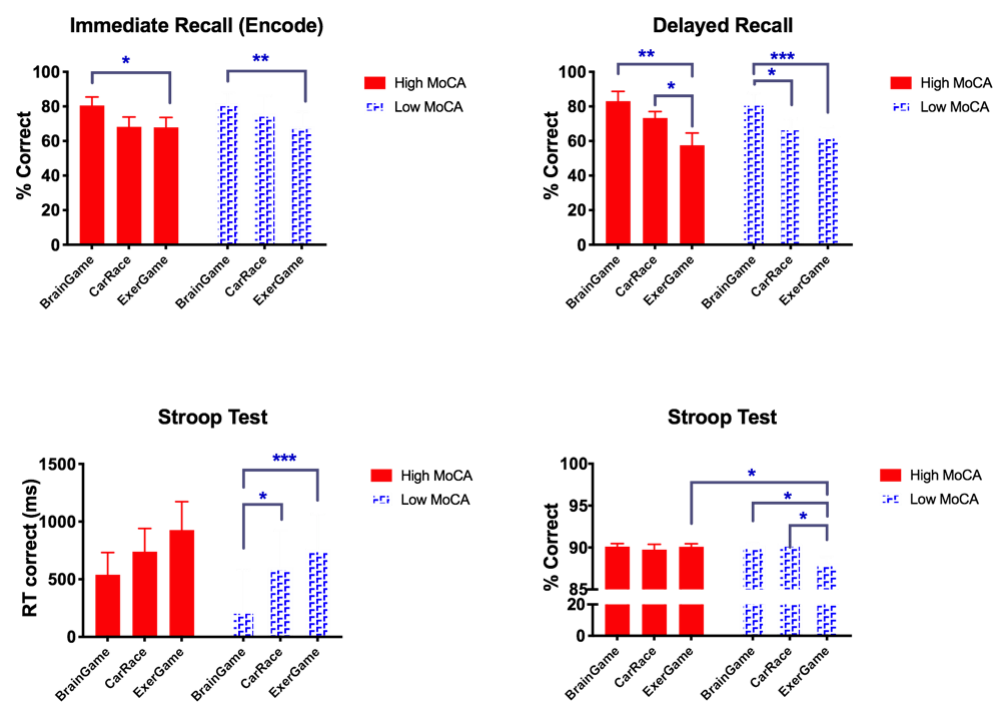
Differences in cognitive performance between sessions and Montreal Cognitive Assessment groups (*P&lt;.05; **P&lt;.005; ***P&lt;.001). MoCA: Montreal Cognitive Assessment.

We had hypothesized that if a game was stressful, then it would interfere with cognitive processing. Indeed, we found that different game *sessions* affected the rate of correct immediate recall in the encode (χ^2^_2_=10.2; *P*=.006) and delayed recall (χ^2^_2_=24.6; *P*<.001), as well as on Stroop’s RT to correct (χ^2^_2_=6.5; *P*<.05) but not on Stroop rate of correct response (χ^2^_2_=2.5; *P*=.29). Post hoc tests showed significant reduction of performance in the exergame session (S_3_) compared with baseline (brain training, S_1_) during encode (mean difference −12.6%; *P*=.02; 95% CI −23.5 to −1.68) and recall (mean difference −22.96; *P*<.001; 95% CI −35.6 to −10.2). A similar trend was observed in comparison of Car Race (S_2_) vs S_1_ (for encode, mean difference −10.9; *P*=.09; 95% CI −23.3 to 1.45; and for recall, mean difference −11.48; *P*=.06; 95% CI −23.4 to 0.47). No difference in encode rates was observed between S_3_ and S_2_, but there was a trend of reduced correct recalls (mean difference −11.48%; *P*=.08; 95% CI −24.0 to 1.06). Stroop RT was also slower in S_3_ vs S_1_ (mean difference 440 ms; *P*=.006; 95% CI 130 to 759) and S_2_ vs S_1_ (mean difference 269 ms; *P*=.03; 95% CI 20 to 510). Because encode and recall rates were correlated (*r=.631*; *P*<.001), we controlled for the effect of encode hit rates in the GEE model that estimated the effect of the session on recall hits. Regardless, the effects of *Session* on recall performance remained significant (χ^2^_2_=16.1; *P*<.001). These results suggest that even though the participants had become familiar with the tasks, their performance did not improve by the third session; it declined instead, suggesting that the exergame was the most challenging and potentially the most stressful game of the three. Figure 5 illustrates how this stress effect on performance was exacerbated in the group with lower MoCA scores.

### Effects of Game Type on Reflective Outcomes

**Figure 6:**
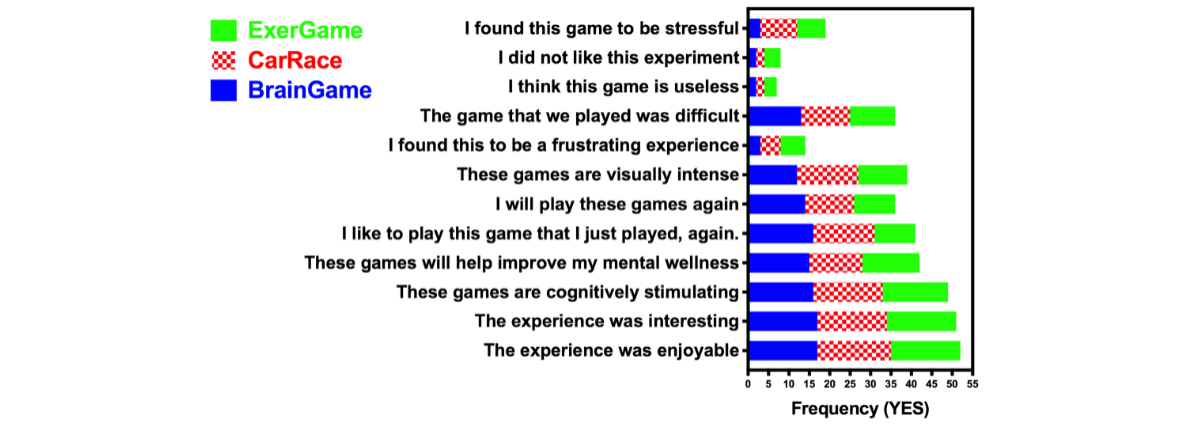
Frequency of responding positively to the postgame appraisal questionnaire at the end of each session.

The experimental sessions did not cause changes in anxiety states as measured by the 6-item Spielberger State-Trait Anxiety Inventory Scale (χ^2^_2_=2.0; *P*=.36). However, participants did express an emotional response to the playing experience, as shown in Figure 6. Because our sample was small, we did not perform any factor analysis. Instead, we chose to illustrate the frequency of affirmative responses to each of the game appraisal questions at the end of the session. Most participants found all three games to be enjoyable, interesting, stimulating to cognition, and beneficial to mental wellness.

We evaluated game-related differences in appraisal of the session using a Kruskal-Wallis comparison of Likert-scale responses to each item. We did not find significant differences related to the experimental session in scoring items. The test statistics are presented in Multimedia Appendix 1. Although we did not perform any factor analysis, we explored the correlation among the variables. It is worth reporting that there was a significant positive correlation between expressing a will to play the game again (*I will play this game again*) and finding the game *Good for Mental Wellness* (Spearman =0.826, n=54; *P*<.001), *Cognitively Stimulating* (Spearman (N=54)=0.545; *P*<.001), and *Enjoyable* (Spearman =0.639, n=54; *P*<.001), confirming the link between meaningfulness and enjoyment. There was also a negative correlation between expressing a *Will to Play the Game again*, and finding it *Frustrating* (Spearman =−0.438, n=54; *P*<.001) and *Useless* (Spearman =−0.617, n=54; *P*<.001). A more detailed analysis of these results is beyond the scope of this report and will be presented separately, together with the qualitative data [89].

### Effects of Cognitive Ability on Outcome Measures

#### Characteristics of the Sample in Montreal Cognitive Assessment Groups

To examine whether cognitive reserve would predict the stressfulness of the gaming experience, the sample was split based on MoCA scores to low-MoCA (MoCA<27; n=8; mean 24.62, SD 1.41) and high-MoCA (MoCA ≥27; n=11; mean 28.27, SD 1.19) groups.

Independent *t* tests did not show significant differences in age (95% CI −3.8 to 2.3; *P*=.53), Perceived Stress Scale (95% CI −3.36 to 4.36; *P*=.73), General Self-Efficacy (95% CI −3.6 to 2; *P*=.53) or Loneliness scale (95% CI −19 to 2; *P*=.13)

#### Montreal Cognitive Assessment Group Differences in Game Appraisal

**Figure 7:**
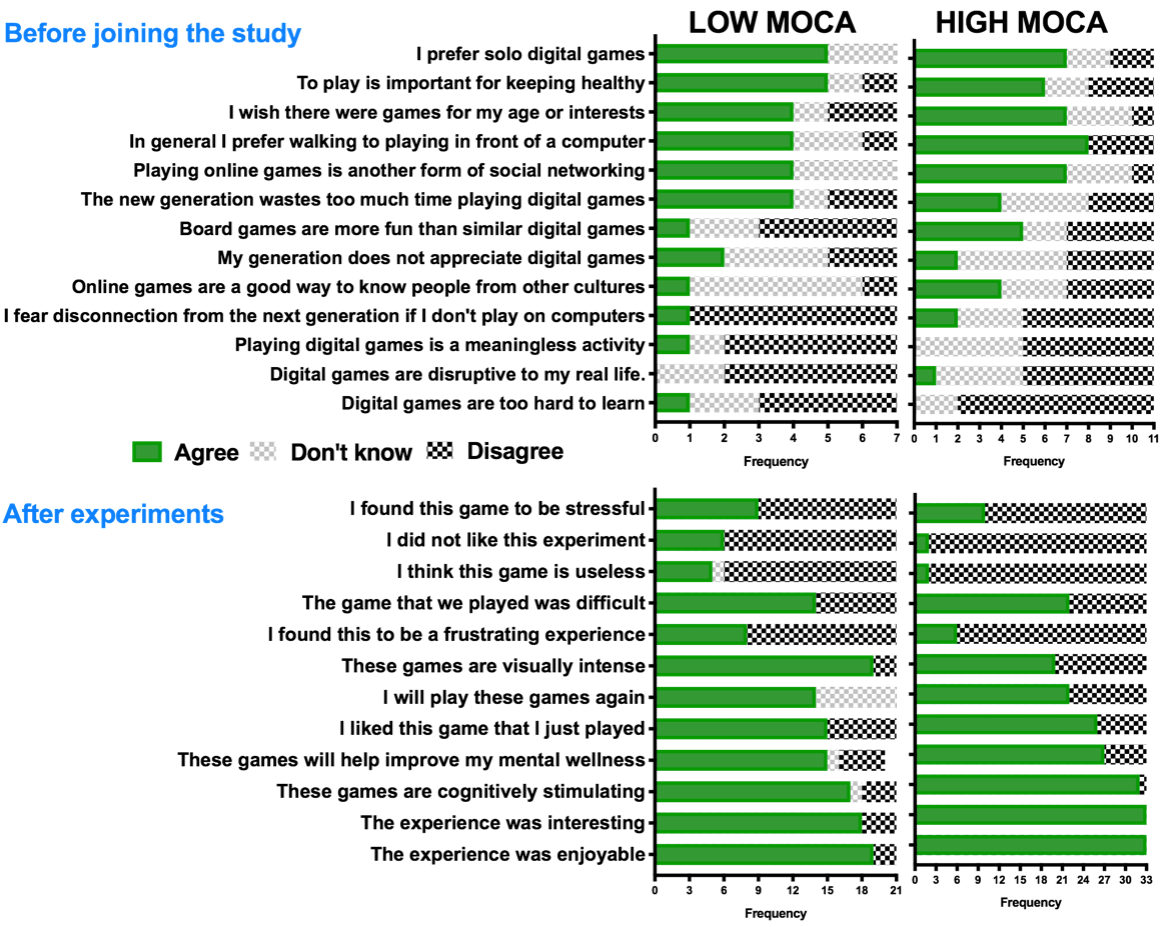
Response frequencies to the pre-enrollment and the postgame appraisal questionnaires.

Figure 7 illustrates the patterns of game appraisal in the MoCA groups. In the screening questionnaires, the high-MoCA groups had a generally more positive attitude toward playful experience. The Kruskal-Wallis test demonstrated that in the pre-enrollment appraisal questionnaire, the low-MoCA group considered digital games *too hard to learn* (χ^2^_1_=4.05; *P*<.05), but other scores were not significantly different. In the postgame appraisal questionnaires, the low-MoCA group disliked the experience more (χ^2^_1_=5.1; *P*<.03) and found the game more useless (χ^2^_1_=4.92; *P*<.03), less interesting (χ^2^_1_=4.90; *P*<.03), and visually more intense (χ^2^_1_=5.6; *P*<.02); no other differences were statistically significant.

#### Montreal Cognitive Assessment Group Differences in Physiological Response

**Figure 8:**
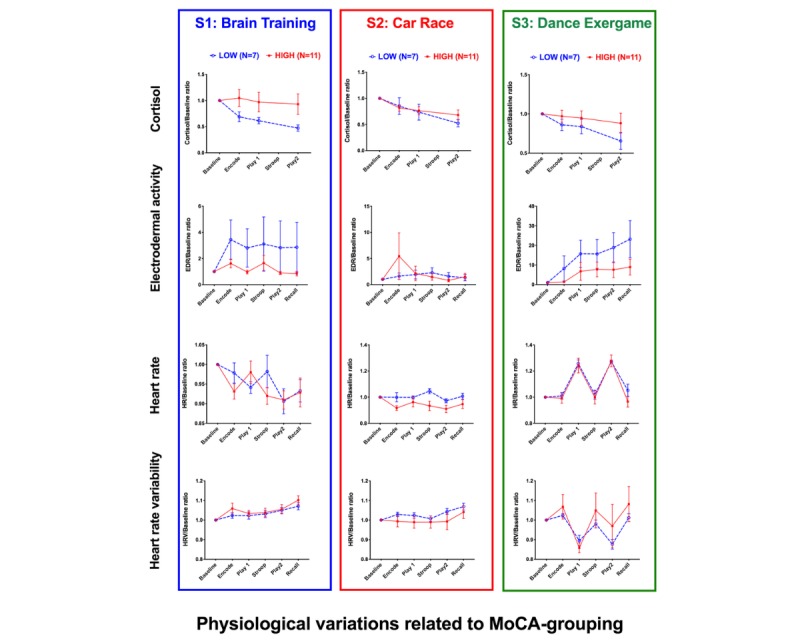
Group×activity interaction effects. All results shown are normalized to the baseline (within-subject, and within-session) and plotted as mean+standard error of the mean. MoCA: Montreal Cognitive Assessment.

We expected individuals with lower MoCA scores to find the games more challenging and therefore be more stressed by it. To compare the groups, we first normalized all the physiological variables to the within-session and within-subject baseline. Figure 8 illustrates group differences in the pattern of physiological signals over time. Because the physiological profile was significantly different between sessions, we compared group differences separately for each session. A GEE model with independent variable MoCA was then tested for each physiological dependent variable. The results are summarized in Multimedia Appendix 1. The only significant differences in reflexive responses to the experiment were observed in the cortisol levels in the first session (S_1_) and in the HR during the CareRace (S_2_).

In S_1_, cortisol in the low-MoCA group had a significantly larger drop from the baseline (mean difference −0.218; *P*=.046; 95% CI 0.004 to 0.432). This effect can reflect the fact that the low-MoCA group was more stressed about taking part in the study from the onset. Although not statistically significant, this group showed a steeper decline in cortisol level in the exergame session as well.

In S_2_, the low-MoCA group increased their HR with respect to baseline and compared with the high-MoCA group. A trend for increased HR during cognitive tests during S_1_ (Encode and Stroop) was also observed in the first session. These effects might suggest that irrespective of games, the low-MoCA group found the cognitive challenges in the experiment difficult and stressful.

#### Montreal Cognitive Assessment Group Differences in Cognitive Tests

We expected that the performance of the low-MoCA group in cognitive tasks would be significantly reduced compared with that of the high-MoCA group. As Figure 5 illustrates, the interactions between *MoCA Group* and *Session* were mainly driven by session effects, which affected both groups similarly. The GEE test (*Session*_i_×MoCA) revealed significant interaction effects on all cognitive variables (all *P*<.005), except Stroop Correct (χ^2^_5_=7.4; *P*=.19). Post hoc analyses show that for both groups, performance in memory tasks was significantly lowered in S_3_ vs S_1_ (12% less hits in immediate recall, *P*<.001; and 22% less hits in delayed recall, *P*<.001). The Stroop RT was significantly increased in S_3_ vs S_1_ (mean difference 461 ms; *P*=.001). In the low-MoCA group, a small reduction in Stroop accuracy was observed between S_3_ and S_1_ (mean difference −2.1%; *P*<.03) and between S_3_ and S_2_ (mean difference −2.33%; *P*=.008). The Stroop RT to correct in S_3_ was also the highest and significantly different from that of S_1_ (mean difference 535 ms; *P*<.001)*.* The only significant difference between the two groups was in percentage of correct Stroop in S_3_ (mean difference 2.03%; *P*<.03). Although the game difficulty affected the Stroop performance in the low-MoCA group, it did not have any impact on the performance of the high-MoCA group.

## Discussion

### Summary

This study presents a prototype for AGPHA, which incorporates quantitative methods from stress research, to design serious games for use by older adults. The theoretical basis of AGPHA considers appraisal as the first criterion for the evaluation of gamified strategies for seniors’ health care. We performed a mixed methods study to investigate whether playing unfamiliar digital games in the context of their cognitive benefits would be stressful for older adults. We measured stress both reflectively (using appraisal questionnaires before enrollment and after each game-play session), and reflexively (using a wearable device to measure electrodermal response, HR, and HRV; as well as from saliva cortisol). We found an overall positive attitude toward games and a significant link between general cognitive abilities (measured using MoCA, a clinical instrument that measures short-term memory, orientation, visuospatial and executive functions, language abilities, abstraction, and attention) and the degree to which different games (especially the more difficult one, exergame) affect the game-related physiological and cognitive responses. In the following sections, we discuss the main findings, the questions that arise from this research, and the limitations to overcome in future work.

### Principal Findings

#### What Are the Characteristics of Those Who Participated

Our snowball sampling survey was important in informing the results about the characteristics of those who found the topic of serious games interesting. Interindividual variations in game perception and motivation are important factors that engender the gameplaying experience [58,59]. The AGPHA framework [60] builds on the appraisal theory of stress and coping [90]. When confronted with a new challenging encounter, the primary appraisal process is to categorize it as irrelevant, benign-positive, or *stressful*, depending on what implications it would have for the individual’s well-being. If the person has no investment in the outcome of the challenge, then they will have no need for it and will not commit to engaging with it. On the contrary, if they perceive immediate or potential benefits, they are willing to try it and enter the secondary appraisal stage. It is in the second stage that the individual focuses on evaluating the challenge: *Is it feasible and within my physical cognitive abilities?*

Our recruitment advertisement targeted a large mailing list of people interested in preventive health care and attracted 42 responses to the survey. Therefore, the number of individuals who entered the primary appraisal stage was not large. Of those, only 19 agreed to enter the secondary appraisal process. Those who did not participate were more likely to agree with the statement *Digital games are too hard to learn*, suggesting that from the onset, those with less confidence about their cognitive abilities excluded themselves from the experimental phase. Overall, the patterns of response (Figure 3) illustrated that the participating group had a generally more positive attitude toward gaming activities. The participating and nonparticipating groups did not differ in any other characteristics (eg, age, education, self-efficacy)

It must be emphasized that the participating sample had high self-efficacy and was well educated and socially and cognitively active. Thus, the following discussion hinges on the fact that the participants in the study were not representative of the aging population who are in need of cognitive or physical assistance. To address their needs by gamified digital interventions, we must repeat this experiment in a group with pronounced cognitive or physical disabilities (eg, in a nursing home or a rehabilitation center). Theoretically, any factors (such as accessibility or executive difficulty) that influence game appraisal will theoretically change the subsequent reflective and reflexive outcomes as well.

#### Are New Gaming Experiences Stressful for Seniors?

The second question we asked was whether playing a new digital game would cause stress. If this were the case, then we would expect to observe a significant increase in cortisol and ambulatory signals after the introduction of each game. As the patterns of signal in Figure 4 illustrate, this was not the case.

Cortisol is the hallmark of a latent psychosocial response to stress, but we did not see any significant cortisol effect after any of the gaming activities in the experiment. One explanation is that the highest levels of cortisol were measured at arrival time. This could have resulted from the participant’s anticipation of the session, or from exertion of walking to our center. Because the hypothalamic pituitary adrenal stress system is regulated through a negative feedback mechanism, it is plausible that while the body was downregulating the high levels of basal cortisol, the system was not as responsive to the stress that may have been introduced during game play [91]. Detrending the cortisol signal to remove the sharp decay showed only a mild (and statistically insignificant) increase in cortisol post exergame.

While the cortisol response results from complex and relatively slow neuroendocrine processes, the EDA and HRV mark sympathetic autonomic response and have widely been used in human-computer interface studies [76-82]. The exergame was physiologically more demanding and higher physiological response was expected. Indeed, we observed significant differences in EDA, HR, and HRV, which significantly changed during the exergame (Figure 4) independent of the effect of movement and body temperature (measured on E4).

Interestingly, reflexive and reflective responses were not corresponding. Despite being more difficult, the exergame was not subjectively rated as more stressful (Figure 6). Nevertheless, the lowering of performance in cognitive tasks, which became statistically significant in the stress-sensitive recall task [71], indicates that it had a more pronounced interference with cognitive processing. This interpretation is consistent with previous reports that have shown a link between cognition and training with exergames [92-95]. In other words, the fact that exergame affects the cognitive performance immediately proves that it exercises the brain. Given that effects of stress on cognition are mediated through reward processing [83] and attention [84] systems, the challenge for game designers as well as gerontologists is to evaluate to what extent, for whom, and which exercises can become beneficial to the neurological health of aging individuals.

#### Effect of Cognitive Abilities on Stress Response to Games

Our third question was whether differences in cognitive ability (based on MoCA) predicted differences in reflective (Figure 7) and reflexive (Figure 8) response to games.

In terms of primary appraisal, the low-MoCA group found *digital games* *too hard to learn*. In the secondary appraisal (ie, after playing), the low-MoCA group was less likely to *enjoy* the game or find it *interesting*, less likely to find it *useful*, and more likely to find the game *visually more intense*. However, the groups did not differ in finding the games *difficult* or *stressful*. According to the appraisal theory, these anticipatory and perceptual differences should predict an increased stress response.

Indeed, physiological reactions to the games were stronger in the low-MoCA groups, although they were not statistically significant. The exception was in cortisol levels. The low-MoCA group had 21% more decrease in cortisol levels with respect to baseline during the first session (brain training). This effect may have resulted from the anticipatory stress of the first session, or from the MoCA test itself, which was administered before the experiment began, and possibly caused a social evaluative stress.

Despite differences in perception and cortisol dynamics, cognitive performance in the first session was not significantly different between the two groups, and they both had high rates of correct responses to cognitive tests (>80%). However, in both groups, the performance in those tasks was lowered by increasing game difficulty and more pronounced in the low-MoCA group (Figure 5).

Van Reekum et al [80] have demonstrated the correlation between autonomic responses (cardiac and electrodermal signals) and performance in an action video game. It is plausible that the low-MoCA group experienced higher states of arousal as a result of adaptive coping to compensate for their initial discomfort with the game. This interpretation is consistent with the observation of Birks et al [58], who have argued that adding extrinsic motivation increases the efforts of those who do not have high levels of affinity with their game avatar. Thus, an increased stress response in the low-MoCA group could indicate the player’s increased engagement with the game. Whether this engagement will lead to a desire to repeat play needs to be tested.

### Comparison With Previous Studies

#### Intergenerational Considerations

To the best of our knowledge, this is the first quantitative crossover study to have compared the psychophysiological responses of older adults to different genres of marketplace games, with different levels of complexity in aesthetics, dynamics, and mechanics of games. Intergenerational differences in such studies must address differences in needs and values, as well as differences in physiology.

Poels et al [79] used a similar study design, but their participants were 19 young players. In the study by Poels et al, participants were monitored for hedonic and physiological reactions to four action games with different aesthetic features (two first-person shooters and two race games, with varying degrees of visual complexity). Their study indicated that initial physiological reactions predicted the likelihood of repeat playing in the long term.

Mandryk and Atkins [81] found that galvanic skin response, facial electromyograms, and cardiovascular responses can be used in machine learning algorithms to dynamically compute the degrees of valence and arousal during a gameplay session in 24 young gamers. Their study showed a high convergence between the subjective ratings of games and the machine-predicted levels of emotion and arousal.

In our study, postgame appraisal suggests that the desire to play the game again was highest in the brain training game (14 of 18 players indicated that they will play those games again), which was associated with lower levels of physiological response. In other words, it appears that younger players in the study by Poels et al [79] played games to induce a physiological response, whereas older players in our study preferred a game that did not induce strong reflexive responses.

Our findings are consistent with previous reports that in general, more seniors prefer the ease and pleasure of casual games (preferably with an intellectual component) over more cognitively demanding action games [3,56,57]. Our observations raise the following important questions that cannot be addressed in this study. What are the intergenerational differences in game appraisal? How are these differences in game appraisal correlated to age-related differences in physiological and cognitive reserves? Are physiological measures appropriate for evaluation of seniors desire to engage in the game challenge?

#### The Importance of Context

In a meta-analysis of 48 studies, involving uniplayer games (excluding exergames), Van der Vijgh et al [96] demonstrated and argued that although games can explain up to 57% of stress-related physiological variations (HR and blood pressure), it is not the game alone but the context of the experimental design and the study characteristics that moderate the stressful response to games.

In this study, we wanted to make the participants comfortable with games (eg, by sequencing the games from simple in the first session to hard in the last session). However, these games were presented in a *serious* context by the title of the study *An Objective Assessment of Interactions Between Appraisal, Arousal and Cognitive Benefits of Electronic Playing*. If the study characteristics (which we modified by altering the games in the different sessions) are important, then arguably, our complex experimental setting, and the inclusion of the memory and Stroop tests (even though simplified) could have altered the phenomenology of the experience and thus have hindered the physiological responses to the gameplay alone. Nevertheless, our complex experimental setup allows to illustrate the complexity arising from contextual factors that interact with psychophysiological adaptation. In real life, games are not experienced as isolated laboratory events, but in relation to other activities. The complexity of Group×Session×Activity interactions with physiological signals (Figure 8) provides evidence to concur with Van der Vijgh et al [96] that it is the context in which games are experienced that counts.

#### Stress and Performance

Acute stress is not necessarily a maladaptive response. Stress interacts with learning and cognitive function through myriad physiological and behavioral cascades [64,84]. The evolutionary function of an acute physiological stress response is to focus and consolidate the lessons to be learned from surviving new challenges [65,84,97,98].

Van Reekum et al [80] have demonstrated a correlation between autonomic responses (cardiac and electrodermal signals) and performance in an action video game. In our design, neither could we reliably record nor did we want to measure the player’s performance in the commercial games that we presented. Instead, we used the simple version of a Stroop test in which RT is expected to be affected by acute stress [71,86], and which seems to acutely improve after casual exergaming in a younger sample [87]. We also used an encoding/recall task, which we have previously shown to be sensitive to acute stress [71,85]. Although these measures were not chosen to be difficult (eg, the number of Stroop trials was very small) or to measure specific cognitive domains, they served as control variables to help us assess whether game difficulty could have an impact that exceeded the learning effect.

We found that the RT to a very simple Stroop (4 color, 75% incongruent stimuli) increased with game difficulty and that delayed recall accuracy decreased with game difficulty, especially in those with lower MoCA scores. Although we cannot infer about how games interacted with cognition, we demonstrate that such cognitive tests may offer a lower cost measure to trace effects of repeated play on neuropsychological factors that underlie cognition. As we have argued in introduction of the AGPHA framework [60], to document such data can potentially guide the decisions made in the design cycle, in terms of how to activate specific cognitive targets by modifying game aesthetics and dynamics.

### Limitations

#### Sample Size

This study is limited primarily by the sample size, which was estimated with the expectation of 80% of power to detect a cortisol response to an explicit stress challenge [71]. In older adults, stress responses to the same task may be blunter than those in a younger sample [99]. Age-related variations in stress sensitivity may be connected to a myriad of endocrinological or psychological factors, all of which need to be carefully modeled using larger samples [100]. Adding multifactorial quantitative measurements (growing in availability and reducing in cost) to larger crowdsourced experiments such as the one conducted by Birks et al [101] could provide a finer grained picture of the physiological embodied qualities of a game experience. This would be of considerable importance for the clinical industries that are emerging around the utilization of game-based interventions in mental health care [32].

In addition, the complexity of our setup makes our ambulatory measurements more susceptible to instrumentation noise. Despite setting up the procedure to be identical and obtaining repeated measures of cortisol and ambulatory signals at baseline, we did not achieve interclass correlations above 70%. Endocrine measurements (eg, cortisol) are sensitive to factors such as time of the day (circadian rhythms), activity before sampling, and states of health and medications. Although we controlled and measured all of these factors, our small sample size does not allow including all of them in our statistical models. These factors must be controlled for in larger studies.

#### Experimental Complexity and Analytical Limits

The second limitation of this study is inherent to its experimental design. We wanted these experiments to be participatory and gave primacy to the participant’s appraisal of games in relation to their cognitive benefits. We therefore avoided stressful elements such as keeping scores or time pressure. The main experimental variants were gradual increase in game complexity and MoCA categorization. Our repeated measures experimental design is advantageous in terms of statistical power but is not immune to habituation effects, which is a very important and confounding factor in stress studies.

The third limitation of our study is to have split the sample by MoCA scores. MoCA includes 30 questions, and a score of 1/0 per each question, to account for differences in short-term memory, orientation, visuospatial and executive functions, language abilities, abstraction, and attention. This categorization, especially in the context of evaluating a complex and interactive medium such as a game, is too reductionist. To use MoCA as a discriminating factor provided methodological simplicity to offer a proof-of-concept example of the application of AGPHA framework, but it did not account for the variations in behavioral strategies that compensate for decline in those specific cognitive domains. These issues must be further investigated.

Finally, our test studio (blue-floored and mirrored-walls) and the various assessment tools we used were quite burdensome and far from ecological. To repeat this experiment in a familiar setting and with a simpler design, such as in a living laboratory, a nursing home, or a senior’s community center, will likely produce different results.

### Conclusion and Future Work

The field of serious games needs methodological standards to evaluate the efficacy of games that are designed to benefit seniors [50]. Despite its limitations, this pilot study illustrates that the quantitative framework proposed in AGPHA is sensitive to uncovering the within-subject and between-group differences in reflexive and reflective reaction to games. We have used various instruments: surveys and appraisal inventories (which are available free of cost), multiple data from wearable physiological monitors (the device costs ~Can $2500 [~US $1871]), multiple saliva samples (~Can $15 [~US $11.22813] per each sample), and computerized neurocognitive tests (almost free). We found a complex pattern of associations between physiological factors and activities in each session, which suggests that variables such as cortisol and slow EDA signal changes are sensitive for detecting gross effects of experimental procedures. More simple measures—a delayed picture recall test and the RT to a correct response in a nonchallenging version of the Stroop test—were also sensitive enough to detect between-group and between-session variations in response to different games. Better defined cognitive tests can reveal more precise interactions between specific games and specific cognitive domains. The MoCA categorization in this study serves as a proof of concept for the AGPHA framework, but it does not represent the full scope of interindividual variations (eg, in sex, gender, personality, and health state). In larger and more heterogeneous samples, this multivariate approach can be explored from multiple facets to help us develop predictable models of health outcomes benefiting from different serious games.

## Appendix

Multimedia Appendix 1 Supplemental materials.

## Abbreviations

ACC: accelerometer
AGPHA: Affective Game Planning for Health Applications
EDA: electrodermal activity
GEE: generalized estimating equation
HR: heart rate
HRV: heart rate variability
MoCA: Montreal Cognitive Assessment
RT: reaction time
