# Supplementary material for: Reflective and Reflexive Stress Responses of Older Adults to Three Gaming Experiences In Relation to Their Cognitive Abilities: Mixed Methods Crossover Study
Source: JMIR Ment Health. 2020 Mar 26;7(3):e12388. doi: 10.2196/12388 (PMC7146255; doi:10.2196/12388)
Supplement: Multimedia Appendix 1 [file mental_v7i3e12388_app1.docx]

**Multimedia Appendix 1**

**TABLE OF CONTENTS**

[Details of Experimental Procedure 1](#_Toc28866062)

[Baseline 1](#_Toc28866063)

[Session 1: 2](#_Toc28866064)

[Session 2: 2](#_Toc28866065)

[Session 3: 2](#_Toc28866066)

[Table S1: Kruskal-Wallis test of effect of game appraisal at the end of each session. Means are calculated over the range of responses to the listed question (-2, definitely disagree; 2, definitely agree). 2](#_Toc28866067)

[Table S2: GEE tests of relation between MOCA and physiological factors 4](#_Toc28866068)

## Details of Experimental Procedure


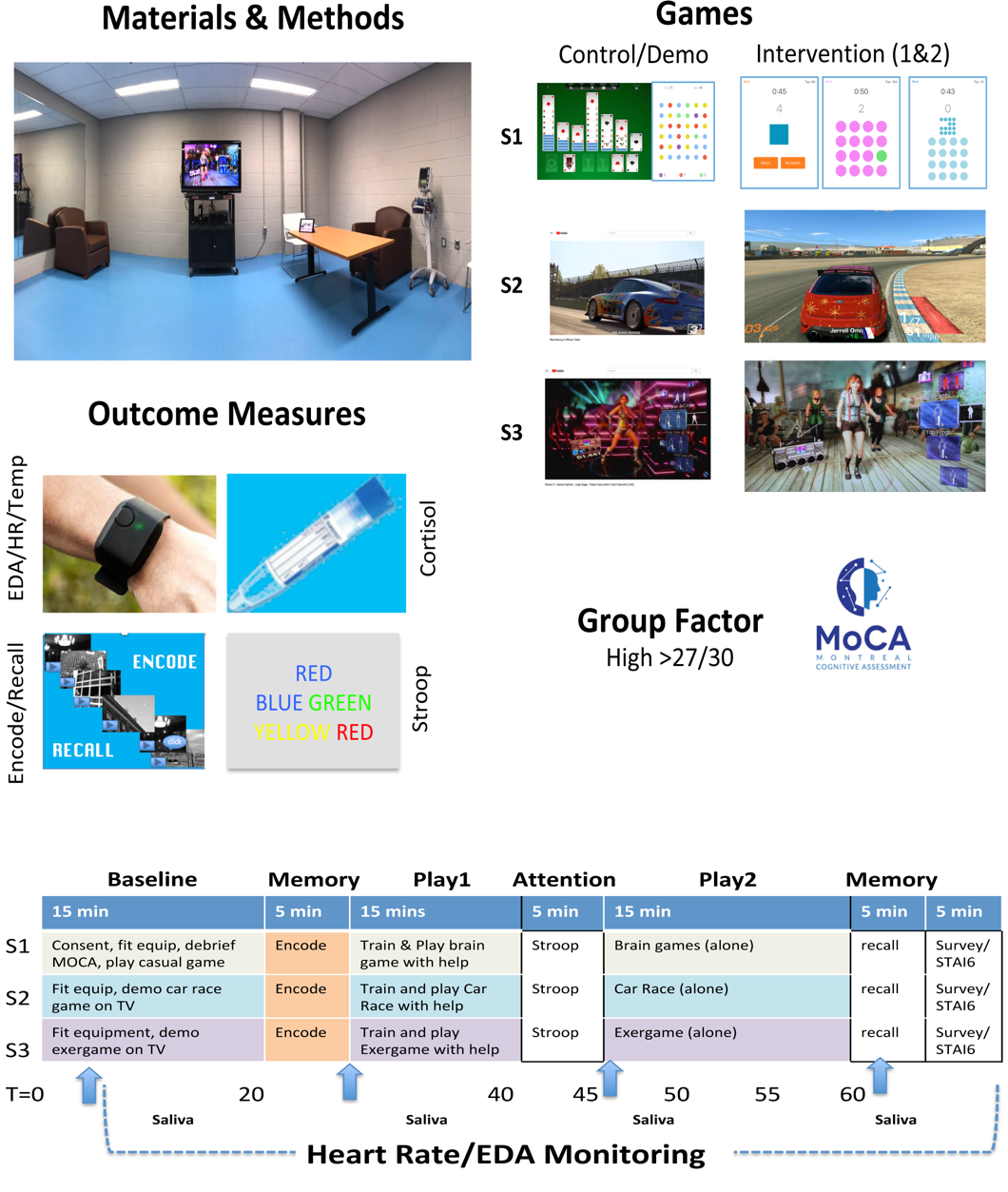


Baseline *(performed at the beginning each three visits)*: Upon arrival, participants were briefed about the procedure, provided a saliva sample, were fitted with an EDA/HR monitor (E4, Empathica Inc.). Only in session one, we administered the MOCA and the 8UG test, to obtain the baselines of cognitive and physical fitness. We then talked for about 5 minutes about the games they would be playing and the rationale for choosing each game (as explained below). One saliva sample was collected after the briefing and before starting the encode test which was administered on a laptop. A STAI-6 questionnaire was administered at the beginning of each session to assess subjective rating of anxiety about the experiment. The following games were then administered in each session:

Session 1: The aim of this session was to introduce players to cognitive training games, and to compare the psychophysical reaction to these games against casual, non-serious games. Participants received a 10-minute demo of casual games, *Solitaire* (v1.5.6, Brainium Studios LLC, for iPad) and *Dots* (v 2.3.5, Playdots, Inc.), a free tile-matching game with minimalist design features, aesthetically similar to our cognitive training *MindGames* (v2.5, by Tom Lake)—from which we chose *Reaction, Match and Patterns (Figure 2)*. After the demo, they played each of the *MindGames* for about three minutes each; then performed the CW Stroop Test on a laptop. We then gave them about 15 minutes to play any of the games they learned during the demo and to score the level of fun and difficulty (0-10). At the end, a saliva sample was collected, and the recall task was administered on a laptop.

Session 2: The aim of this session was to introduce a visually and cognitively more complex game, using the same device, iPad. We selected a free car racing game (*Real Racing 3,* *v 5.4.0, Electronic Arts Inc.)* as it is a simple game that allows players to steer the car using the gyroscopic features of the iPad, and therefore it requires minimal efforts in learning the control buttons. During Demo, we first showed the game using its commercial video (<https://www.youtube.com/watch?v=nEmc53kZPMY&t=349s>) by a skilled ‘driver’, on a 42 inch TV monitor, while an assistant explained the rules of the game by pointing to the TV screen. During the practice, the assistant loaded the game for them in the *Amateur* mode, showed them how to steer (by tilting the Tablet) and brake (by pressing the screen), and let them play the game of speed (to completed four laps in a racetrack, and be awarded and ranked based on their finishing speed determined by their ability to stay on-track and avoid collisions). During the free-play session, the assistant loaded the game of elimination, which required the players to finish a short track by ranking lower than the third place, else be eliminated and have to restart the game. During the free play session, the assistant was present but at a distance and left the players to play the game for 15 minutes. Saliva sampling and cognitive tests were administered similarly to session 1, as shown in Figure 1.

Session 3: The aim of this session was to introduce the most complex and sophisticated form of digital game, which promises mental and physical health advantages: an exergame. We chose *Dance Central* (Harmonix, MS Studios), a rhythmic Exergame developed for X-Box360+Kinect. In this game the players copy the moves of a virtual choreographer while the motion tracking Kinect evaluates them against the queued movement. The Kinect camera captures motion and scores players based on the accuracy of their movements. We chose a simple dance routine (*Poker Face*), and showed the commercial video if the game (<https://www.youtube.com/watch?v=IZ95rWRSZD8>) during the demo. This routine was chosen to ensure the safety of players, as it does not have any jumps, or intensive movements on the knee and hip joints of the players. During the training, an assistant set the game to *easy/break it down* mode and played together with a participant to ensure they learned the game for the first 5 minutes, and stayed close by to guide them as they continued playing for another 5 minutes. During the free play, the game was set to *easy/perform it* mode, and the participants were asked to play up to 15 minutes. We recommended to the participants to stop if for any reason they felt physical or psychological discomfort with the game. Saliva sampling and cognitive tests were administered similarly to session 1, as shown in Figure 2.

## Table S1: Kruskal-Wallis test of effect of game appraisal at the end of each session. Means are calculated over the range of responses to the listed question (-2, definitely disagree; 2, definitely agree).

| **Descriptive Statistics** | Mean | Std. Deviation | Mean Ranks | | | Chi-Square (df=2) | Asymp. Sig. |
| --- | --- | --- | --- | --- | --- | --- | --- |
|  |  |  | Brain Game | CarRace | Exergame |  |  |
| I found this game to be stressful | -0.69 | 1.386 | 22.89 | 33.56 | 27.83 | 4.63 | 0.10 |
| I did not like this experiment | -1.45 | 1.303 | 26.39 | 27.86 | 29.83 | 1.03 | 0.60 |
| I think this game is useless | -1.35 | 1.174 | 26.32 | 29.22 | 28.56 | 0.49 | 0.78 |
| The game that we played was difficult | 0.45 | 1.425 | 26.16 | 30.19 | 27.75 | 0.66 | 0.72 |
| I found this to be a frustrating experience | -1.04 | 1.465 | 23.5 | 30.36 | 30.39 | 3.11 | 0.21 |
| These games are visually intense | 0.69 | 1.426 | 25.66 | 33.11 | 25.36 | 3.02 | 0.22 |
| I will play these games again | 0.71 | 1.583 | 30.76 | 27.06 | 26.03 | 1.05 | 0.59 |
| I like to play this game that I just played, again. | 1.07 | 1.399 | 31.74 | 27.03 | 25.03 | 2.27 | 0.32 |
| These games will help improve my mental wellness | 1.06 | 1.338 | 30.32 | 25.56 | 26.36 | 1.16 | 0.56 |
| These games are cognitively stimulating | 1.27 | 1.008 | 27.82 | 29.75 | 26.44 | 0.48 | 0.79 |
| The experience was interesting | 1.75 | 0.865 | 28.16 | 27.92 | 27.92 | 0.01 | 1.00 |
| The experience was enjoyable | 1.67 | 0.818 | 30.89 | 26.64 | 26.31 | 1.84 | 0.40 |

## Table S2: GEE tests of relation between MOCA and physiological factors

|  | **Cortisol** | **EDA** | **HR** | **HRV** |
| --- | --- | --- | --- | --- |
| Model factor | **Wald χ^2,^ df, p** | **Wald χ^2,^ df, p** | **Wald χ^2,^ df, p** | **Wald χ^2,^ df, p** |
| MOCA (High - Low) | .84, 1, .359 | .148, 1, .700 | 1.75, 1, .186 | 1.67, 1, .197 |
| MOCA * Session | 6.08, 4, .193 | 22.17, 4, <.001 | 183, 4, <.001 | 16.53, 4, .002 |
| MOCA * Activity | 20.34, 4, <.001 | 323, 10, <.001 | 298, 10, <.001 | 277, 10, <.001 |
|  |  |  |  |  |
